# Global access to affordable direct oral anticoagulants

**DOI:** 10.2471/BLT.20.278473

**Published:** 2021-06-01

**Authors:** Ignacio Neumann, Holger J Schünemann, Lisa Bero, Graham Cooke, Nicola Magrini, Lorenzo Moja

**Affiliations:** a Department of Internal Medicine, Pontificia Universidad Católica de Chile, Santiago, Chile.; b Department of Health Research Methods, Evidence and Impact, McMaster University, Hamilton, Canada.; c Center for Bioethics and Humanities, University of Colorado Anschutz Medical Campus, Aurora, United States of America.; d Department of Infectious Diseases, Imperial College London, London, England.; e Italian Medicines Agency, Rome, Italy.; f Secretariat of the Model List of Essential Medicines, Department of Essential Health Products and Standards, World Health Organization, Avenue Appia 20, 1211 Geneva 27, Switzerland.

## Abstract

Poor control of cardiovascular disease accounts for a substantial proportion of the disease burden in developing countries, but often essential anticoagulant medicines for preventing strokes and embolisms are not widely available. In 2019, direct oral anticoagulants were added to the World Health Organization’s *WHO Model list of essential medicines*. The aims of this paper are to summarize the benefits of direct oral anticoagulants for patients with cardiovascular disease and to discuss ways of increasing their usage internationally. Although the cost of direct oral anticoagulants has provoked debate, the affordability of introducing these drugs into clinical practice could be increased by: price negotiation; pooled procurement; competitive tendering; the use of patent pools; and expanded use of generics. In 2017, only 14 of 137 countries that had adopted national essential medicines lists included a direct oral anticoagulant on their lists. This number could increase rapidly if problems with availability and affordability can be tackled. Once the types of patient likely to benefit from direct oral anticoagulants have been clearly defined in clinical practice guidelines, coverage can be more accurately determined and associated costs can be better managed. Government action is required to ensure that direct oral anticoagulants are covered by national budgets because the absence of reimbursement remains an impediment to achieving universal coverage. Tackling cardiovascular disease with the aid of direct oral anticoagulants is an essential component of efforts to achieve the World Health Organization’s target of reducing premature deaths due to noncommunicable disease by 25% by 2025.

## Résumé

L'absence de lutte efficace contre les maladies cardiovasculaires contribue grandement à la charge de morbidité pesant sur les pays en développement. Pourtant, les anticoagulants essentiels permettant d'éviter les accidents vasculaires cérébraux et les embolies sont souvent difficiles à obtenir. En 2019, les anticoagulants oraux directs ont été ajoutés à la Liste modèle des médicaments essentiels publiée par l'Organisation mondiale de la Santé. Le présent document vise à résumer les avantages des anticoagulants oraux directs pour les patients souffrant d'une maladie cardiovasculaire, et à évoquer les moyens d'encourager leur utilisation au niveau international. Bien que le coût des anticoagulants oraux directs ait fait débat, intégrer ces médicaments dans la pratique clinique les rendrait plus abordables grâce à diverses méthodes: négociation des prix; achats groupés; appels d'offres concurrentiels; communautés de brevets; et recours accru aux alternatives génériques. En 2017, seulement 14 des 137 pays ayant adopté des listes nationales de médicaments essentiels y avaient inclus des anticoagulants oraux directs. Ce chiffre pourrait augmenter rapidement si les problèmes de disponibilité et d'accessibilité peuvent être résolus. Dès que les profils des patients susceptibles d'être traités par des anticoagulants oraux directs sont clairement établis dans les directives de pratique clinique, la couverture peut être définie avec plus de précision et les dépenses correspondantes, mieux gérées. Les gouvernements doivent s'assurer que ces médicaments sont bien pris en compte dans les budgets nationaux, car l'absence de remboursement demeure un obstacle à la couverture maladie universelle. La lutte contre les maladies cardiovasculaires à l'aide des anticoagulants oraux directs est un élément essentiel des efforts destinés à atteindre l'objectif de l'OMS: faire baisser de 25% d'ici 2025 les décès prématurés dus aux maladies non transmissibles de 25% d'ici 2025.

## Resumen

El mal control de las enfermedades cardiovasculares representa una proporción importante de la carga de enfermedades en los países en desarrollo, y a menudo los medicamentos anticoagulantes esenciales para prevenir los accidentes cerebrovasculares y las embolias no son fácilmente accesibles. En 2019, los anticoagulantes orales directos se añadieron a la lista modelo de medicamentos esenciales de la Organización Mundial de la Salud. Los objetivos del presente artículo son resumir los beneficios de los anticoagulantes orales directos para los pacientes con enfermedades cardiovasculares y discutir las formas de aumentar su uso a nivel internacional. Aunque el coste de los anticoagulantes orales directos ha suscitado debate, la asequibilidad de introducir estos medicamentos en la práctica clínica podría aumentarse al: negociar precios; hacer adquisiciones conjuntas; hacer licitaciones competitivas; utilizar consorcios de patentes; y ampliar el uso de genéricos. En 2017, solo 14 de los 137 países que habían adoptado listas nacionales de medicamentos esenciales incluían un anticoagulante oral directo en sus listas. Este número podría aumentar rápidamente si se pueden abordar los problemas de disponibilidad y asequibilidad. Cuando los tipos de pacientes que pueden beneficiarse de los anticoagulantes orales directos se hayan definido claramente en las directrices de la práctica clínica, la cobertura podrá determinarse con mayor precisión y los costes asociados podrán gestionarse mejor. Es necesario que los gobiernos actúen para garantizar que los anticoagulantes orales directos estén cubiertos por los presupuestos nacionales, ya que la ausencia de reembolso sigue siendo un impedimento para lograr la cobertura universal. La lucha contra las enfermedades cardiovasculares con la ayuda de los anticoagulantes orales directos es un componente esencial de los esfuerzos por alcanzar el objetivo de la OMS de reducir las muertes prematuras debidas a enfermedades no transmisibles en un 25 % para 2025.

## ملخص

إن ضعف التحكم في أمراض الأوعية الدموية هو المسؤول عن نسبة كبيرة من عبء المرض في البلدان النامية، إلا إن غالبًا ما تكون الأدوية المضادة للتخثر والأساسية للوقاية من السكتات الدماغية وانسدادات الأوعية الدموية، غير متوفرة على نطاق واسع. في عام 2019، تمت إضافة مضادات التخثر الفموية المباشرة إلى القائمة النموذجية للأدوية الأساسية التابعة لمنظمة الصحة العالمية. تهدف هذه الورقة إلى تلخيص فوائد مضادات التخثر الفموية المباشرة بالنسبة للمرضى المصابين بأمراض الأوعية الدموية، ومناقشة طرق زيادة استخدامها دوليًا. على الرغم من أن تكلفة مضادات التخثر الفموية المباشرة قد أثارت الجدل، إلا أن إمكانية طرح هذه العقاقير للممارسة الإكلينيكية، يمكن أن تزداد من خلال: التفاوض على الأسعار؛ والمشتريات المجمعة؛ وطرح المناقصات التنافسية؛ واستخدام مجمعات براءات الاختراع؛ والتوسع في استخدام الأدوية العامة المكافئة. في عام 2017، قامت 14 دولة فقط بتضمين مضادات التخثر الفموية المباشرة في قوائمها، من بين 137 دولة اعتمدت قوائم الأدوية الأساسية الوطنية. ويمكن لهذا الرقم أن يزداد بسرعة إذا أمكن التعامل مع مشاكل التوافر والتكاليف المعقولة. بمجرد أن يتم تحديد أنواع المرضى المحتمل أن يستفيدوا من مضادات التخثر الفموية المباشرة بوضوح في إرشادات الممارسة الإكلينيكية، يمكن تحديد التغطية بدقة أكبر، ويمكن إدارة التكاليف المرتبطة بها بشكل أفضل. هناك حاجة لإجراء حكومي لضمان تغطية مضادات التخثر الفموية المباشرة بواسطة الميزانيات الوطنية، وذلك لأن غياب التمويل يظل كعائق في سبيل تحقيق التغطية الشاملة. إن التعامل مع أمراض الأوعية الدموية بمساعدة مضادات التخثر الفموية، يمثل مكونًا أساسيًا في جهود تحقيق هدف منظمة الصحة العالمية، والمتمثل في الحد من الوفيات في سن صغيرة بسبب الأمراض غير المعدية بنسبة 25% بحلول عام 2025.

## 摘要

对心血管疾病控制不力的主要原因在于发展中国家的疾病负担过重，但是往往又无法广泛提供预防中风和栓塞的基本抗凝药物。2019 年，直接口服抗凝药被列入世界卫生组织的《世卫组织基本药物标准清单》。本文旨在概述直接口服抗凝药对心血管疾病患者的益处，并讨论在国际上推广使用该药物的方法。尽管直接口服抗凝药的成本引发了争论，但为了将这些药物引入临床实践流程，可通过以下方式提高这些药物的可负担性：商议价格；集中采购；竞争性招标；使用专利池；以及增加非专利药物的使用量。2017 年，在采用国家基本药物清单的 137 个国家中，只有 14 个国家将直接口服抗凝药纳入其清单之中。如果能够解决可获得性和可负担性相关问题，则该数量可能会迅速增加。如果临床实践指南明确定义了直接口服抗凝药的潜在受益患者类型，则可更准确地确定覆盖范围并更好地管理相关费用。必须获得政府支持，以确保直接口服抗凝药被列入国家预算清单，否则不能报销费用仍然是实现全民覆盖的一大阻力。使用直接口服抗凝药治疗心血管疾病是实现以下世卫组织目标的重要工作之一：截至 2025 年将非传染性疾病导致的过早死亡率降低 25％。

## Резюме

Неэффективные меры по борьбе с болезнями сердечно-сосудистой системы являются причиной проблем, связанных с контролем соответствующих заболеваний в развивающихся странах, однако широкий доступ к основным антикоагулянтам для предотвращения инсультов и эмболии часто отсутствует. В 2019 году пероральные антикоагулянты прямого действия были добавлены в Примерный перечень ВОЗ основных лекарственных средств. Цели данной статьи — кратко изложить преимущества пероральных антикоагулянтов прямого действия для пациентов с сердечно-сосудистыми заболеваниями и обсудить пути расширения их использования на международном уровне. Хотя стоимость пероральных антикоагулянтов прямого действия вызвала дискуссию, доступность внедрения этих препаратов в клиническую практику может быть расширена за счет переговоров о цене, комплексных закупок, конкурсных торгов, использования патентных пулов и расширенного использования непатентованных лекарственных препаратов. В 2017 году лишь 14 из 137 стран, принявших национальные перечни основных лекарственных средств, включали в свои перечни пероральные антикоагулянты прямого действия. Это число можно быстро увеличить, если удастся решить проблемы, связанные с наличием и доступностью. После того как в руководящих принципах по клинической практике будут четко определены типы пациентов, которым пероральные антикоагулянты прямого действия могут быть полезны, можно будет более точно определить охват и более эффективно управлять соответствующими расходами. Правительства должны обеспечить покрытие расходов на пероральные антикоагулянты прямого действия за счет национальных бюджетов, поскольку отсутствие компенсации остается препятствием для достижения всеобщего доступа к соответствующим препаратам. Борьба с сердечно-сосудистыми заболеваниями с помощью пероральных антикоагулянтов прямого действия является важным компонентом усилий по достижению цели ВОЗ, направленной на сокращение преждевременной смертности от неинфекционных заболеваний на 25% к 2025 году.

## Introduction

In April 2019, the World Health Organization’s (WHO) Expert Committee on the Selection and Use of Essential Medicines recommended that dabigatran – a direct oral anticoagulant – should be added to the core list of essential medicines.^1^ Dabigatran was listed with a square box symbol, which indicates that specified alternatives (e.g. apixaban, edoxaban and rivaroxaban) have full therapeutic equivalence at all approved doses. The decision was based on two independent applications for direct oral anticoagulants: (i) one for their use in individuals with atrial fibrillation;^2^ and (ii) the other for their use in individuals with atrial fibrillation or venous thromboembolism.^3^ This was the second time an application for the inclusion of direct oral anticoagulants had been considered.^4^ In 2015, however, the expert committee rejected the application and commented that further research was required on the unmet need for anticoagulation, particularly in low- and middle-income countries, and that the large cost difference between direct oral anticoagulants and warfarin was disproportionate to the incremental benefit observed.

It is, therefore, timely to reflect upon the arguments presented to WHO’s expert committee on the potential public health impact of including direct oral anticoagulants on national essential medicines lists and to consider how the number of patients who could benefit can be rapidly increased. Listing a medicine as essential is the first step in determining which medicines a country should stock, prescribe and dispense. Moreover, the listing increases the chance that higher-priced essential medicines will be reimbursed by national governments, thereby giving patients easier access to life-saving medicines they cannot afford to buy. At the same time, countries could face disastrous economic consequences without some form of price control.

In 2010, an estimated 33.5 million people worldwide had atrial fibrillation.^5^ A study of strokes in 15 400 patients with atrial fibrillation in 47 countries in 2016 found that the annual incidence was greatest in Africa, at 8% (89/1137), compared with 7% (143/2023) in China and 7% (88/1331) in South-East Asia.^6^ Venous thromboembolism is also common: in 2014, the incidence ranged from 0.7 to 2.7 per 1000 patient-years in Western Europe, from 1.1 to 2.4 per 1000 patient-years in North America and from 0.2 to 1.6 per 1000 patient-years in Latin America and Asia.^7^

Anticoagulation therapy has been associated with a 64% reduction in the risk of stroke in people with nonvalvular atrial fibrillation and an 80% reduction in the risk of recurrent venous thromboembolism in those with deep venous thrombosis or pulmonary embolism.^8^^,^^9^ The narrow therapeutic window (i.e. the safe and effective dose range) of vitamin K antagonists makes it difficult to achieve optimal anticoagulation. In one European study, for example, the proportion of patients with poorly controlled treatment varied from 35% (935/2702) in the United Kingdom of Great Britain and Northern Ireland to 56% (673/1208) in Germany.^10^ Among low- and middle-income countries, the proportion of patients with poor anticoagulation has been reported to be as high as 78% (461/588) in Latin America and 83% (1581/1899) in Asia.^11^ As could be expected, poor anticoagulation is associated with worse outcomes and higher overall mortality.^11^

## Merits of direct oral anticoagulants

A new medicine is rarely found that is both more effective and safer than a highly effective comparator, and that also has a good level of supporting evidence. However, direct oral anticoagulants are one example. These drugs can be used as alternatives to vitamin K antagonists in individuals with atrial fibrillation and an intermediate or high risk of stroke, as well as in patients with venous thromboembolism. Data from large randomized trials indicate that direct oral anticoagulants have a better safety profile than vitamin K antagonists and that they are equally effective, though there is probably little difference between the two in absolute mortality. Table 1 and Table 2 summarize data from comprehensive evidence syntheses from randomized trials that compared direct oral anticoagulants with vitamin K antagonists.^3^^,^^12^ Table 1 shows clinical outcomes in patients with nonvalvular atrial fibrillation and Table 2 shows outcomes in those with a deep vein thrombosis or pulmonary embolism. Several other systematic reviews have reached similar quantitative and qualitative conclusions.^13^^–^^15^

**Table 1:** Treatment outcomes with direct oral anticoagulants versus vitamin K antagonists in patients with nonvalvular atrial fibrillation, meta-analysis, 2019

Outcome	No. of trial participants followed up^a^	Evidence quality^b^	Relative risk (95% CI) of outcome with direct oral anticoagulants versus vitamin K antagonists	Incidence of outcome in first year of treatment	
				With vitamin K antagonists, per 1000 patients	Difference between direct oral anticoagulants and vitamin K antagonists, per 1000 patients (95% CI)
Death	73 641	High	0.90 (0.85 to 0.94)	75	−7 (−11 to −4)
Stroke	75 543	High^c^	0.83 (0.72 to 0.96)	33	−6 (−9 to −1)
Systemic embolism	75 018	Moderate^d,e^	0.74 (0.48 to 1.13)^f^	3	−1 (−1 to 0)
Major bleeding	75 490	Moderate^g^	0.81 (0.66 to 0.98)	59	−11 (−20 to −1)

CI: confidence interval.

^a^ The meta-analysis included participants in 13 randomized controlled trials.

^b^ Evidence quality was assessed using the grading of recommendations assessment, development and evaluation (GRADE) approach.

^c^ Although some heterogeneity was observed (*I^2^* = 47%), we did not downgrade the level of evidence because of inconsistency.

^d^ Although some heterogeneity was observed (*I^2^* = 31%), we did not downgrade the level of evidence because of inconsistency.

^e^ The level of evidence was downgraded because of imprecision (i.e. studies included few patients and few events and, therefore, the confidence interval for the effect was large).

^f^ As the confidence interval probably crosses decision thresholds, the possibility of either benefit or harm cannot be excluded.

^g^ Significant heterogeneity was observed (*I^2^* = 77%).

Data source: Neumann & Schünemann, 2019.^3^

**Table 2:** Treatment outcomes with direct oral anticoagulants versus vitamin K antagonists in patients with deep vein thromboses or pulmonary embolisms, meta-analysis, 2020

Outcome	No. of trial participants followed up^a^	Evidence quality^b^	Relative risk (95% CI) of outcome with direct oral anticoagulants versus vitamin K antagonists		Incidence of outcome in first year of treatment	
					With vitamin K antagonists, per 1000 patients	Difference between direct oral anticoagulants and vitamin K antagonists, per 1000 patients (95% CI)
Death	28 778	Moderate^c^	0.99 (0.85 to 1.15)^d^		39	0 (−6 to 6)
Pulmonary embolism	28 571	Moderate^c^	0.97 (0.77 to 1.23)^d^		20	−1 (−5 to 5)
Proximal deep vein thrombosis	28 668	Moderate^c^	0.80 (0.59 to 1.09)^d^		26	−5 (−11 to 2)
Major bleeding^e^	28 876	High	0.63 (0.47 to 0.84)		17	−6 (−9 to −3)

CI: confidence interval.

^a^ The meta-analysis included participants in 12 randomized controlled trials.

^b^ Evidence quality was assessed using the grading of recommendations assessment, development and evaluation (GRADE) approach.

^c^ The level of evidence was downgraded because of imprecision (i.e. studies included few patients and few events and, therefore, the confidence interval for the effect was large).

^d^ As the confidence interval probably crosses decision thresholds, the possibility of either benefit or harm cannot be excluded.

^e^ At 6 months.

Data source: Ortel et al., 2020.^12^

As vitamin K antagonists have a narrow therapeutic window and highly variable pharmacokinetics, their use requires strict medical follow-up and dose-monitoring, as well as lifestyle and dietary changes. In contrast, direct oral anticoagulants have more predictable pharmacokinetics and can be administered without dose-monitoring. Although the causes of, and potential solutions for, medication nonadherence are complex, direct oral anticoagulants have the advantage that their use is simple for patients, prescribers and the health-care system. Moreover, these drugs are increasingly used for bridging anticoagulation therapy in patients undergoing surgery or other invasive procedures and have replaced low-molecular-weight heparin during the perioperative period.^16^

In 2015, WHO’s expert committee on essential medicines was reluctant to include direct oral anticoagulants on the list of essential medicines, partly because an antidote was unavailable. Recently, however, idarucizumab and andexanet alfa have been approved as reversal agents for dabigatran, rivaroxaban and apixaban,^17^^–^^19^ though their efficacy and cost–effectiveness remain uncertain in some settings and patient populations.^20^ Fortunately, bleeding is rare. For instance, in the ROCKET trial,^21^ the rate of life-threatening bleeding in patients with atrial fibrillation was 0.8 and 1.2 per 100 patient-years in the rivaroxaban and warfarin groups, respectively. Current guidelines recommend cessation of the direct oral anticoagulant and supportive care in such cases but do not recommend the routine use of reversal agents.^22^

Even in highly controlled settings, such as direct oral anticoagulant experimental trials, where patients are properly monitored and those on vitamin K antagonist are maintained in optimal anticoagulation, study participants were in the therapeutic international ratio range for only 60 to 70% of the time.^23^ Given the difficulty of achieving optimal anticoagulation with vitamin K antagonists in routine clinical practice, then, the medium-term effects of introducing direct oral anticoagulants may be greater than observed in current trials. Data from observational studies seem to confirm this possibility and, in addition, indicate that direct oral anticoagulants also perform better than vitamin K antagonists in older, and more severely ill, patients treated outside of clinical trials.^2^

### Patients’ preferences

A systematic review of patients’ preferences for direct oral anticoagulants or warfarin, which were determined using either a survey or the discrete choice method, found that patients preferred direct oral anticoagulants because they were convenient to use and involved less lifestyle modification.^24^ This preference can also be observed in the progressive increase in direct oral anticoagulant uptake over the years and the accompanying reduction in vitamin K antagonist use. For example, data from the GARFIELD registry,^25^ which was a longitudinal study of individuals from 50 countries with newly diagnosed, nonvalvular atrial fibrillation, show that the proportion of anticoagulant therapy involving direct oral anticoagulants increased from 4% (224/5500) to 37% (4065/11 046) between 2010 and 2015 and that the proportion involving vitamin K antagonists decreased from 53% (2815/5311) to 34% (3714/10 923). Nevertheless, in general, patients who were well controlled on vitamin K antagonists and who had no complications preferred to remain on these compounds rather than to switch to direct oral anticoagulants.

In making its recommendation, WHO’s expert committee on essential medicines noted that the underlying philosophy of anticoagulation therapy was to offer a choice of options that included, in particular, treatments that do not depend on frequent laboratory testing. Moreover, as the provision of several anticoagulant therapies involves an opportunity cost for health services, the committee was particularly careful in making its decision. The committee requested that all evidence available in 2015 and afterwards should be reviewed before coming to a final decision in 2019 when it became clear that patients were being deprived of a beneficial treatment.

## Increasing access

Despite the global burden of death and disability linked to heart disease, access to the medicines essential for preventing and treating cardiac syndromes remains challenging. There are substantial barriers to anticoagulation therapies, especially in low-resource settings. For instance, adherence to long-term therapy is often poor and tends to decrease over time in patients with non- or pauci-symptomatic conditions. Governments play a central role in improving access to essential medicines for circulatory diseases and in ensuring that all patients in need receive safe and effective treatment. In addition, the widespread use of direct oral anticoagulants in the future, as part of universal health coverage, will depend on developing a competitive market.

### Price

Since direct oral anticoagulants first entered the market, they have been priced substantially higher than vitamin K antagonists. However, although this price differential may impact out-of-pocket expenditure, it could have a smaller effect on overall costs. Patients receiving vitamin K antagonists require dose-monitoring and frequent visits to anticoagulation clinics. From the payer’s perspective, then, the availability of an anticoagulation monitoring system is a prerequisite for safely providing anticoagulation with vitamin K antagonists. In contrast, direct oral anticoagulants do not require dose-monitoring and the frequency of follow-ups is dictated by the patient’s health rather than by the need for dose adjustments. Hence, when the cost of running anticoagulation clinics or their equivalents is considered, the direct costs of vitamin K antagonists and direct oral anticoagulants are likely to be similar.

Recent systematic reviews of economic evaluations have moved from assessing willingness to pay from a fixed-budget perspective and have adopted a payer’s perspective. As a result, reviews now indicate that it is cheaper to anticoagulate patients with nonvalvular atrial fibrillation or venous thromboembolism using direct oral anticoagulants than vitamin K antagonists.^26^^,^^27^ Furthermore, the pharmaceutical market can change rapidly: once generic alternatives are available, competition and pricing negotiations can reduce the financial burden for countries. Table 3 illustrates how prices for direct oral anticoagulants can vary globally and puts potential target pricing in context. For example, the monthly cost of dabigatran and rivaroxaban in Brazil was as low as 20 United States dollars (US$), which was 50 to 95% below prices in some other countries. Given current price trends, we forecast that the cost of generic direct oral anticoagulants can easily be far less than US$ 1 per day (for two tablets) in most countries.

**Table 3:** Estimated cost of direct oral anticoagulants, by WHO Region and country, 2019

Region and country	Approximate monthly cost (US$)		
	Dabigatran	Rivaroxaban	Apixaban
**Americas**			
Argentina	50	150	ND
Brazil	20	20	ND
Canada and United States	300 to 601	300 to 601	300 to 601
Chile	30	65	ND
Colombia	30	65	ND
**European**			
United Kingdom of Great Britain and Northern Ireland^a^	90	90	90
**South-East Asia**			
India	61	57	61
**Western Pacific**			
Australia	65	60	68
China	222	742	370

ND: not determined; US$: United States dollar.

^a^ In the United Kingdom of Great Britain and Northern Ireland, edoxaban also cost approximately 90 United States dollars per month.

### Introduction into the health-care system

In 2017, the cost of direct oral anticoagulants was reimbursed in several high-income countries, whereas only a few low- and middle-income countries recognized their potential advantages and listed them beside vitamin K antagonists in their national essential medicines lists.^28^ In 2019, dabigatran was listed by 14 countries out of 137 investigated: Bahrain, Bulgaria, Croatia, Czechia, Estonia, the Islamic Republic of Iran, Mexico, Poland, Portugal, the Russian Federation, Slovakia, Slovenia, Sri Lanka and Sweden.^29^ Ten countries listed another therapeutically equivalent direct oral anticoagulant (e.g. rivaroxaban and apixaban) as an alternative. However, in many places where private and public health-care provision coexist, direct oral anticoagulants have been preferentially introduced in the private sector, thereby increasing gaps in health equity. For example, a recent observational study of prescription patterns in individuals with atrial fibrillation in the United States of America found that the probability of receiving a direct oral anticoagulant instead of a vitamin K antagonist was higher in educated individuals with a high income.^30^

Health authorities need to develop a strategic model for managing the introduction of direct oral anticoagulants into their health-care systems and for avoiding serious discrepancies in access. Strategies should include extensive prelaunch activities, risk-sharing arrangements, restrictions on prescribing, and monitoring prescribing after direct oral anticoagulants have been introduced.^31^ In the past, several countries experienced difficulties with managing the launch of direct oral anticoagulants: they subsequently withdrew them from reimbursement lists, restricted access or struggled for funding.^31^

### Improving usage

For individual countries, the use of direct oral anticoagulants is usually defined by clinical practice guidelines, which determine who should be prescribed the drugs. Countries with low resources or where direct oral anticoagulants are expensive might restrict their use to high-risk populations. In several countries, these drugs were initially used as non-routine treatment for highly selected patients who were ineligible for vitamin K antagonists. However, active marketing by drug companies encouraged extending their use to patients who might not have been eligible for direct oral anticoagulants according to local guidelines or local medical authorities. In Australia, direct oral anticoagulant-related events sponsored by the pharmaceutical industry reached 90 000 health professionals over a period of 4 years.^32^ By favouring expensive alternatives or unnecessary drug use, promotional marketing can undermine the principle behind essential medicines lists, which is the careful selection of safe and effective medicines. Government agencies should promote independent information on direct oral anticoagulants and control the quality of continuous medical education.

Although thresholds for an increased risk of stroke may vary between regions, individuals with atrial fibrillation and one risk factor for stroke are generally considered to have an intermediate risk (estimated annual risk: 3%), whereas those who have had a previous stroke or who have two or more risk factors are defined as high risk (estimated annual risk: 8%).^33^^,^^34^ People who have had a venous thromboembolism provoked by a transient risk factor, such as recent surgery, are considered to have a low risk of recurrence (estimated annual risk of a new event: 4%), whereas those who experience an unprovoked event are generally considered at a high risk (estimated annual risk: 7%).^35^ Treating high-risk individuals with direct oral anticoagulants is a starting point for achieving the 50% treatment coverage threshold for eligible individuals but policies are needed to extend treatment to those with an intermediate risk. This 50% coverage threshold is a voluntary target of WHO’s global action plan for noncommunicable diseases and is a precondition for reducing premature deaths due to heart attacks and strokes.^36^

### Pooled procurement

With pooled procurement, the needs of several buyers are aggregated, which influences price negotiations with manufacturers. In Chile, for example, the public health-care system covers approximately 85% of the population and, although the administration of health-care facilities is decentralized, many medications are purchased centrally. With this mechanism, the cost of dabigatran to the public system is approximately 60% of its price at pharmacies (I Neumann, personal communication, December 2020). However, Chile is a relatively small country. The purchasing power of bigger countries may be larger and could further reduce the cost of direct oral anticoagulants. Overall, countries should avoid low levels of pooled procurement or a high degree of procurement fragmentation. The efficiency of procurement is maximized when demand can be predicted with high confidence and contract payments are reliable. These factors will decrease transaction costs and the market risk for suppliers, thereby encouraging better offers and preferential prices. 

In the *WHO Model list of essential medicines*, direct oral anticoagulants are listed with the square box symbol, without a preferred formulation being specified for individual indications.^37^ This indication was done because all approved doses of different formulations are valid alternatives. Moreover, it enables countries or central health-care hubs to choose to purchase a single formulation that covers all doses required or to make selective purchases and limit the use of alternative formulations to common indications, thereby achieving the best tendering prices.

### Generic alternatives

Generic direct oral anticoagulants are expected to become more available in the next 5 years. The market exclusivity period has ended for dabigatran, the first direct oral anticoagulants approved by the United States Food and Drug Administration (FDA; October 2010), and generic alternatives have already been introduced in some countries.^38^ In 2019, the FDA approved the first generic versions of apixaban and, in 2020, the European Medicines Agency approved generic versions of rivaroxaban.^39^^,^^40^

The introduction of generic bioequivalents to market jurisdictions is subject to local legislation and approval from national authorities. Product regulation and market access can be affected by a lack of transparency or by collusion between manufacturing companies and political or technical bodies.^41^ Although switching from an original drug to its generic is still debated in some countries, the evidence shows that there are no clinically meaningful differences from the reference product in terms of quality, safety or efficacy and that generics should be considered as therapeutically equivalent for procurement purposes.^37^^,^^42^ Placing an unnecessary burden and cost on the development and licensing of generics can delay the availability of alternative drugs and affect national competitive pharmaceutical markets. As a result, the level of local competition can vary greatly between countries, as illustrated by the cost differences in direct oral anticoagulants presented in Table 3. Explicit or tacit agreements between companies to set prices above market-clearing rates, thereby leading to a cartelized market, are also possible, particularly when there are few competitors, as is the case for direct oral anticoagulants. Governments are the primary coordinators of efficient tendering processes and should ensure market competition is enforced by expanding the pool of potential suppliers.

### Patent pools and prequalification

Improving access to novel medicines requires new strategies. One emerging approach is the use of patent pools, which enable third parties to acquire nonexclusive licences for the intellectual property needed to market medicines. These pools can help address some of the barriers to access for low- and middle-income countries. The Medicines Patent Pool is a public health organization backed by the United Nations that negotiates medicine licences with patent holders. Historically, negotiations have concerned the treatment of communicable diseases (e.g. human immunodeficiency virus infection). In 2018, the Medicines Patent Pool extended its mandate to include other patented essential medicines.^43^ One product category that could potentially be licensed through the patent pool was identified by analysing the epidemiology, treatment landscape, market size and pricing of direct oral anticoagulants in low- and middle-income countries. The Medicines Patent Pool estimated that, in 2018, public health licensing of direct oral anticoagulants could facilitate up to 1.9 million patient-years of treatment for non-atrial fibrillation and venous thromboembolism.^44^ In addition, patent pools could generate substantial savings for national health systems because direct expenditure on medicines would be calibrated to local budgets by recognized international organizations. Potential savings are in the order of hundreds of millions of US$. Important information on patented small molecule medicines, including dabigatran, apixaban and edoxaban, can be found in MedsPaL,^45^ a free database on the intellectual property status of patented medicines included in the *WHO Model list of essential medicines* for low- and middle-income countries.

Another important contributor to the increased availability and affordability of essential medicines is WHO’s prequalification programme. This service helps local authorities in low- and middle-income countries manufacture, regulate and monitor the quality of medicines considered important for public health.^46^ High-quality generic versions of dabigatran have already been produced and it is possible that the launch of large programmes against noncommunicable diseases could encourage competitive manufacturers to develop other generic direct oral anticoagulants. Where resources are limited, prequalification of a generic should be regarded as a precondition for its selection as an essential medicine. Prequalification could occur simultaneously with public health licensing (with appropriate royalties), thereby creating profitable conditions for medicines to be marketed at a low cost.^47^

## Conclusions

Tackling cardiovascular disease is essential for achieving WHO’s target of reducing premature deaths due to noncommunicable diseases by 25% by 2025.^48^ One step in this process will involve developing strategies to introduce direct oral anticoagulants and other interventions, identifying those patients most likely to benefit in individual countries and increasing the drugs’ affordability, thus mitigating scepticism by potential adopters. Currently the price of direct oral anticoagulants is a major obstacle for health-care systems. However, this obstacle could be removed through multiple actions, including price negotiations, pooled procurement, competitive tendering, patent pools and expanded use of generics. Including direct oral anticoagulants in national essential medicines lists can help increase the proportion of patients who receive optimal anticoagulation and reduce their risk of premature death.
